# Unraveling the individual and interactive effects of climate and competition on branch growth dynamics in *Pinus koraiensis* in Northeast China

**DOI:** 10.3389/fpls.2025.1545892

**Published:** 2025-02-05

**Authors:** Xuehan Zhao, Zheng Miao, Fengri Li, Yuanshuo Hao, Yumeng Jiang, Lihu Dong

**Affiliations:** ^1^ College of Forestry, Northeast Forestry University, Harbin, Heilongjiang, China; ^2^ Key Laboratory of Sustainable Forest Ecosystem Management-Ministry of Education, School of Forestry, Northeast Forestry University, Harbin, Heilongjiang, China

**Keywords:** *Pinus koraiensis*, branch dynamic growth, competition, climate change, forest management

## Abstract

**Introduction:**

The quantitative modeling of dynamic branch growth in Korean pine (*Pinus koraiensis*) and the analysis of the factors influencing branch growth are essential prerequisites for making scientifically sound management decisions in Korean pine plantations. To date, the effects of competition, climate and their interactions on branch growth have been insufficiently investigated. Additionally, limited knowledge exists regarding whether these impacts vary depending on the social status of trees. In the face of the current challenges posed by climate change, accurate information to inform forest management and policy-making is urgently needed.

**Methods:**

We collected 745 branches from 54 sampled trees of Korean pine and, we employed a mixed-effects model to assess the effects of tree variables, competition, climate, and their interactions on branch growth. Furthermore, we simulated branch growth under different combinations of competition and climatic conditions to provide practical and targeted recommendations for Korean pine plantation management.

**Results:**

Our results demonstrate that (1) in addition to branch age, size, and tree height growth, competition, climate, and their interactions significantly improved the branch growth model, with the effects of interactions surpassing the individual effects of climate, which highlights the importance of considering interactive effects; (2) the effects of climate and competition varied depending on the social status of the trees, with dominant and intermediate individuals showing greater sensitivity to competition and climate than suppressed individuals, suggesting that, for future research in this direction, prioritizing sampling of dominant and intermediate individuals would be a cost-effective approach; and (3) owing to the presence of interactions, the influence of climate on branch growth was modulated by competition, suggesting that adjusting competition levels in response to climate stress could lead to desirable branch growth outcomes.

**Discussion:**

Our study underscores the importance of understanding the different sources of variation in branch growth is crucial for advancing our understanding of tree growth and crown dynamics, as well as for formulating sustainable management policies amidst the uncertainties of climate change.

## 1 Introduction

Korean pine (*Pinus koraiensis*) is a keystone species in the zonal climax communities of Northeast China and plays a crucial role in the restoration and protection of degraded forest areas in the country because of its extreme cold tolerance (Jin et al., 2017; Liu et al., 2024). Furthermore, Korean pine has significant ecological and economic value. Its pine nuts are prized for their nutritional and medicinal properties, and its wood, known for its strength and decay resistance, is widely used in furniture, construction, and other industries (Wang et al., 2019; Li et al., 2021; Zhang et al., 2021). In the region, Korean pine plantations account for approximately 5.2% of the total plantation area (Administration, N.F.a.G, 2019). Optimizing pinecone production, timber yield, and quality is a key objective in managing Korean pine forest ecosystems (Jin et al., 2017). Branches serve as the framework for the tree crown, directly affecting wood quality and cone production. Knots from dead branches can degrade timber quality, causing defects such as discoloration and heart rot, which affect the mechanical properties and utilization efficiency of the wood (Hein, 2008; Wang et al., 2015; Liu et al., 2023a). Although substantial research has focused on the static characteristics of branches, such as their size, number, and angle, studies on their dynamic growth remain relatively scarce (Dong et al., 2015; Gao et al., 2022; Zou et al., 2024). Dynamic branch growth has a direct effect on overall tree structure and biomass distribution, especially across different growth stages and environmental conditions (Hu et al., 2020). Compared with static studies, these studies offer insights into the adaptive strategies of Korean pine and provide essential data for silvicultural practices such as thinning and pruning. For example, research on silver birch suggests that regulating branch growth rates through stand density changes can improve wood quality (Mäkinen, 2002). Understanding dynamic branch growth is crucial for more precise growth pattern quantification, supporting forest management and resource assessment. Additionally, integrating branch dynamics models into stand growth and yield systems can simulate branch size at different stages, providing valuable tools for forest management.

Accurate measurement of branch growth requires both precision and ecological relevance; however, the best assessment method is still unclear (Gleason et al., 2018). Branch growth generally occurs in two steps: initiation and biomass accretion (Cline, 1997; Cline et al., 2009). Both qualitative (e.g., new annual shoots) and quantitative (e.g., biomass increase) approaches are often used (Rahman et al., 2014). Qualitative methods focus on detecting new growth, such as bud formation and leaf unfolding (Yuan et al., 2023). Quantitative metrics, such as branch length, diameter, leaf area, weight (dry and fresh), and annual shoot count, reflect resource acquisition and help predict future growth (Wilson, 2000; de Reffye et al., 2012; Kanazawa et al., 2013; Sun et al., 2019). As critical determinants of branch size, branch elongation and thickening are key for forest management, as they influence crown expansion, tree health, and productivity, with monitoring helping optimize thinning and density adjustments (Mäkinen, 2002; Weiskittel et al., 2007; Nicolini et al., 2012; Kaitaniemi et al., 2020; Guo et al., 2023). However, direct measurement of branch growth is often expensive, necessitating the development of efficient growth models. Statistical models are widely used because of their simplicity and predictive power. For example, Deleuze et al. (1996) predicted annual branch length growth using tree height models. Mäkinen (1999) developed a model for branch diameter growth in Scots pine, incorporating variables such as stem radial growth, branch age, and the height−diameter ratio. Ishii et al. (2000) proposed an allometric growth model for old Douglas fir branches to explain the variability in the relationship between branch diameter and length. Overall, these statistical models provide high reliability in addressing growth pattern variations across different species and habitats. Therefore, this study uses statistical models to simulate branch length and diameter changes in relation to tree growth, environmental factors, and competition.

The factors influencing branch growth can be broadly categorized into endogenous and exogenous factors. Extensive research indicates that endogenous factors such as inherent tree characteristics (e.g., branch height, age, height growth) primarily control branch growth (Mäkinen and Hein, 2006; Osada, 2006; Guo et al., 2023). Competition and climate represent the major exogenous factors, with competition being particularly prominent, often overshadowing climate considerations. When branch-related models are constructed, competition indices are frequently employed to estimate the impact of competition on trees under constraints related to nutrient and water availability (Yang and Huang, 2018; Gao et al., 2022). Studies have shown that competition depends on the ability of plant canopies and root systems to adjust their morphology over time, suggesting that competition is influenced not only by current growth status but also by various factors that evolve over time (Sánchez-Salguero et al., 2015). However, in most current research on competition indices, forest structures are predominantly static, which limits our understanding of dynamic competition indices and their relationships with climate-induced branch growth responses. Hence, in our study, we utilize tree ring data to retrospectively reconstruct dynamic competition indices (Weber et al., 2008; Pach and Soberka, 2011), providing specific information on competition at the individual and temporal levels. In addition, we use annual growth increases rather than cumulative growth as the dependent variable to analyze the effects of these factors on branch dynamics. The objective is to assess the influence of climate and competition on growth precisely while avoiding the average effects of the independent variables on the results.

The branching structure of tree crowns is constructed through the repetitive generation of distinct branch units, and multiple studies have confirmed that climate is a key factor influencing crown development (Wang et al., 2022a; Yan et al., 2023). From a physiological perspective, water availability and temperature affect the accumulation of photosynthetic products and the metabolic rates governing branch growth (Shi et al., 2022). Aberrant water and temperature conditions have been shown to result in leaf shedding, branch breakage, and increased mortality rates (Scott et al., 1993; Shao et al., 2011). Currently, there is limited research on branch responses to climate change, with most studies focusing on developmental processes and potential molecular regulatory mechanisms. Although these studies enhance our understanding of plant functional traits and ecological response mechanisms, they do not directly support forest management practices. Therefore, there is an urgent need to elucidate the impacts of climate change on branch growth. Moreover, climatic factors are often incorporated into models using averages, which overlooks deviations caused by extreme weather events. This underscores the need to consider temporal variability when analyzing the effects of climatic factors, as their fluctuations can have delayed effects that extend beyond the immediate growth period (Liang et al., 2023).

Understanding the interaction between climate and competition is crucial for achieving sustainable management practices (Oboite and Comeau, 2020; Liu et al., 2023b). Competition can influence the effects of climate on tree growth at both the stand and individual tree levels (Khansaritoreh et al., 2017; Young et al., 2017). Research on the relationship between competition and climate enables forest managers to adjust competition measures to facilitate long-term forest adaptation to climate change. Previous studies have shown that competition exacerbates drought stress across a wide range of climates and compositional gradients within forests (Gleason et al., 2017). Additionally, under increasingly arid conditions, performance reversals have been observed in species under high competition levels (Madrigal-González et al., 2018). This interaction between competition and climate may exceed the effects of climate alone (Oboite and Comeau, 2020; Wang et al., 2022a). To our knowledge, there is limited research on the interaction between competition and climate at the branch level.

Climate change has been demonstrated to significantly impact cone production, crown structure, and stem growth in Korean pine (Chen et al., 2022; Wang et al., 2022a; Tian et al., 2024). To quantify the effects of climate change on crown dynamics at the branch level and improve the accuracy of wood quality prediction and cone yield estimation, we examined the branches of 54 Korean pine sample trees and the cores of adjacent trees and aimed to (1) develop a branch growth model to assess how overall tree growth influences branch development, specifically by investigating allometric relationships, and (2) explore the effects of climatic factors, competition, and other variables on branch growth. This research not only enhances and updates model systems for optimizing forest management but also improves our understanding of the response mechanisms of Korean pine plantation ecosystems in the context of climate change, providing a scientific basis for adaptive and resource management of plantations (Dong et al., 2015; Jin et al., 2017; Guo et al., 2023).

## 2 Materials and methods

### 2.1 Study area and data collection

The study area is located within the Benxi Manchu Autonomous County Qinghecheng Experimental Forest Farm, Liaoning Province, in northeastern China, spanning from longitude 123°34′ to 125°56′ and latitude 40°49′ to 41°35′. This region experiences a temperate monsoon climate characterized by long, cold, and dry winters; windy and dry springs; brief, humid, and moderately warm summers; and early frosts and cold autumns. The annual average temperature is 10.7°C, with an average frost-free period of 135 days per year. There are 2379.5 annual sunshine hours on average, and the average annual precipitation is 850 millimeters, which mainly occurs from April to September. The predominant soil type is brown soil.

In total, the measurements were conducted in 18 sample plots (0.06 ha in size) across sites with different conditions in 2023. The measurements included the diameter at breast height (*DBH*, defined as 1.3 m above the ground), total tree height (*HT*), height of the first live branch, and crown radius in four cardinal directions for all trees within each plot. The trees within each plot were ranked in descending order of *DBH*, and the cumulative basal area of *DBH* was calculated and divided into five even intervals to ensure an approximately equal cumulative *DBH* area in each group. The quadratic mean diameter for each group was separately calculated, and trees were designated dominant, intermediate, and suppressed trees on the basis of the first, third, and fifth intervals, respectively. Additionally, the means *DBH* and *HT* of the dominant and suppressed trees were calculated on the basis of the mean diameter and height of the six thickest and thinnest trees (100 trees per hectare) in each plot. A total of 18 dominant trees, 18 intermediate trees, and 18 suppressed trees were selected from the aforementioned plots. A summary of branch, tree and stand variables analyzed in this study can be accessed in **Table 1**.

**Table 1:** Symbols, descriptions, and summary statistics of the branch-, tree-, and stand-level competition variables used in the current study.

Category	Variables (acronym, unit)	Mean	Max	Min	SD
Branch level	Branch age (*AGE*, years)	8.61	34.00	1.00	5.77
(N = 745)	Branch height (*BH*, m)	14.53	24.66	2.27	4.54
	Depth of branch into crown (*DINC*, m)	2.80	11.79	0.01	2.05
	Branch length (*BL*, cm)	196.39	667.00	2.00	133.71
	Branch diameter over bark (*BD*, mm)	23.57	72.85	0.99	14.52
	Branch annual length growth (Δ*BL*, cm)	20.83	66.00	2.00	9.74
	Branch annual diameter growth over bark (Δ*BD*, mm)	2.52	12.94	0.01	1.32
Tree level	Tree height (*HT*, m)	17.33	24.92	3.61	4.14
(N = 54)	Annual tree height growth (*Dheight*, m)	0.30	0.87	0.04	0.14
	Diameter at breast height with bark (*DBH_0_*, cm)	27.43	46.33	4.30	7.73
	Annual diameter at breast height with bark growth (Δ*DBH_0_*, cm)	0.51	2.51	0.01	0.30
	Tree age (*TA*, years)	47.98	70.00	16.00	12.75
Stand level	Quadratic mean diameter (*Dg*, cm)	27.13	36.65	9.33	6.04
(N = 18)	Dominant diameter at breast height (*Ddom*, m)	32.93	43.29	14.60	5.97
	Dominant tree height (*Hdom*, m)	19.07	23.08	10.25	2.65
	Site index (*SI*, m)	20.23	22.77	15.54	2.16
	Density of trees (*N*, tree/ha)	1666.15	3531.25	795.77	871.74
Competition Indices	Hegyi competition index (*CI*)	2.03	14.16	0.15	1.77
	Relative diameter (*RD*)	1.01	1.59	0.42	0.20
	Basal area of the stand (*BAS*, m^2^/ha)	27.50	48.74	3.67	8.86
	Basal area of trees that were larger than the subject tree (*BAL*, m^2^/ha)	14.88	44.45	0.00	10.90

The fixed-radius method is a widely employed technique for identifying competitive trees. Previous research has established that a competition radius of 8 meters or less is optimal for delineating competitive trees (Piutti and Cescatti, 1997; Helluy et al., 2020; Zhou et al., 2022). Accordingly, we defined the maximum competition radius as 8 meters, within which trees were categorized as competitive. The size and spatial relationships of these competitive trees to the target tree were subsequently measured and analyzed. In our study, some plots had undergone thinning, which made it difficult to use the current stand conditions within an 8-meter competition radius to accurately represent historical competition and stand density. Consequently, we focused on branch growth data collected five years after thinning under the assumption that, by this time, the canopy had reclosed and the stand had stabilized. By selecting branch growth data from this specific period, we sought to minimize the influence of previous competition and stand density, thereby facilitating the development of models to assess the impacts of climate and competition on branch growth. Using increment borers, core samples were taken at breast height from competitor trees in both east−west and north−south orientations, and tree-ring width measurements were taken using the WinDENDRO tree-ring analysis system (Regent Instruments, Canada) to obtain diameter growth data of competitor trees for calculating dynamic competition factors.

**Figure 1:**
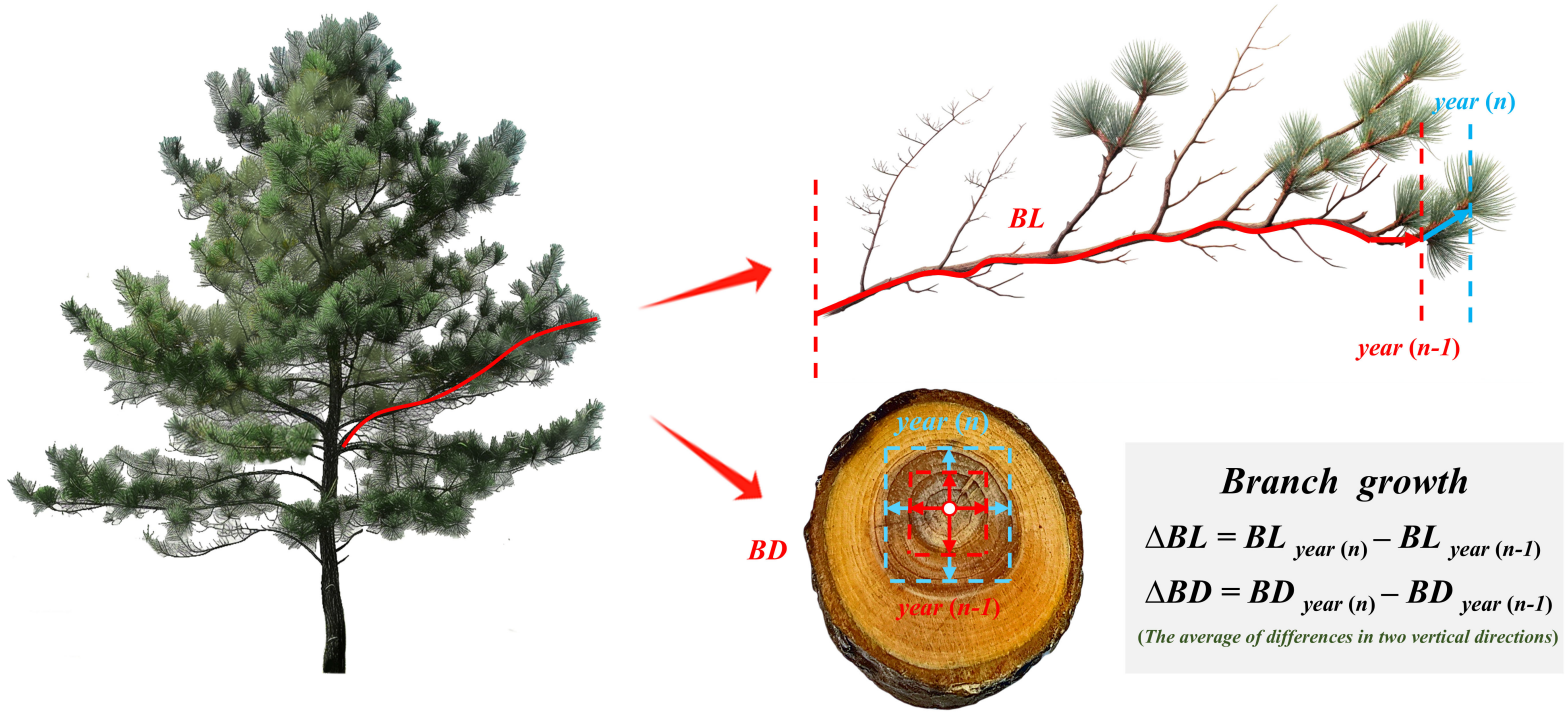
Diagram illustrating annual growth in branch length and diameter.


After a target tree was felled, the total tree height, crown length, and other attributes were measured, and the trunks were segmented at 1-meter intervals. At the stump, at each segment, and at a height of 1.3 m, 3–5 cm thick discs were extracted to obtain breast-height diameter data across different tree ages on the basis of dendrochronological data. Additionally, the attributes of each branch within the live crown were measured: branch status; age; diameter; length; angle; and depth of the branch into the crown (*DINC*). Within each annual growth increment, a medium-sized branch was selected to measure the number of rings, the ring width chronology of the branch base cross-section (measured with a caliper accurate to 0.01 mm), and the length chronology of the branch (measured with a ruler accurate to 1 cm) (**Figure 1**). The growth pattern of evergreen conifers typically involves the production of a main stem, with growth ending annually in a new spiral (with several lateral branches emerging from the same part of the main stem). This indicates a strong correlation between the tree height increment and *DINC*, which allows us to estimate the tree height at different ages by using the average *DINC* of each branch whorl as a proxy for the previous year’s tree height. Furthermore, the diameter at breast height with bark was calculated using a bark coefficient, which has a power function relationship with branch whorls (*AGE*), and the early diameter at breast height for branches (at the target tree’s breast height) was determined as follows:

(1) $$\begin{array}{c} BD(or\;DBH_{0})=K_{B}\cdot BID(or\;DBH_{1})\\ =a\cdot Whrol{\left(or\;AGE\right)}^{b}\cdot BID(or\;DBH_{1}) \end{array}$$

where *BD* and $DBH_{0}$ represent the diameters with bark of branches and trees, respectively; *BID* and *DBH* represent the diameters excluding the bark of branches and trees, respectively; $K_{B}$ is the bark coefficient; whorl and age denote the ages of branches and trees, respectively; and $a_{1}$ =1.34, $b_{1}$ =−0.06, $R_{1}^{2}$ =0.99, $a_{2}$ =1.11, $b_{2}$ =−0.01, and $R_{2}^{2}$ =0.99.

In our study, dynamic competition indices include distance-dependent Hegyi competition indices and distance-independent competition indices (Hegyi, 1974), namely, the common symmetric competition index $\;BA_{ik}\;$ and the asymmetric competition index $BAL_{ik}$ , which reflect competitive relationships (Kuehne et al., 2019).


(2) $$CI_{ik}=\sum_{j=1}^{n_{i}}\frac{d_{jk}}{d_{ik}L_{ij}}$$

where $CI_{ik}$ represents the Hegyi competition index of target tree *i* in year *k*, $n_{i}\;$ denotes the number of competitor trees within the radius excluding the target tree, $d_{ik}$ is the diameter of target tree *i*, $d_{jk}$ is the diameter of competitor tree *j* of target tree *i* in year *k*, and $L_{ij}$ represents the distance between competitor trees *i* and *j*.


(3) $$BA_{ik}=\frac{\pi }{4\cdot S_{compete}}\sum_{j=1}^{n_{i}}D_{jk^{2}}$$

(4) $$BAL_{ik}=\frac{\pi }{4\cdot S_{compete}}\sum_{j=1}^{n_{i}}GD_{jk^{2}}$$

where $BA_{ik}$ and $BAL_{ik}$ denote the basal area (m²/ha) of all stems per hectare and those greater than the sum of the target tree’s basal area for tree *i* in year k, respectively; $GD_{jk}$ represents the diameter of the competitor tree *j* that has a breast height diameter greater than the target tree *i* in year k; and $S_{compete}=\frac{\text{π}}{4}R^{2}$ , where *R* is the competitor radius.

Climate AP serves as a reliable data source, providing readily available information for baseline and future scenarios and yielding promising outcomes in fields such as the growth modeling of forest stands in China, demonstrating its applicability (Wang et al., 2022b; Jiang et al., 2023). The climatic variables for each plot were estimated through spatial interpolation based on longitude, latitude, and altitude. To determine the growth characteristics of Korean pine, annual and seasonal climatic variables (1999–2022) were acquired to analyze their impacts on branch growth. This means that while we conducted a retrospective analysis of branch growth, we also retroactively estimated the climatic variables to match the conditions. Given that tree harvesting occurred from March–April 2023, preceding the growing season, the closest available climate data were from 2022. Furthermore, considering the potential “lag effects” of climate impacts on growth (Tang et al., 2021), climate data were matched from the previous year and two years prior.

### 2.2 Construction of the base model

Korean pine exhibits monopodial growth, characterized by clear annual ring growth in its branches. Branch age is determined by counting rings from the stem tip. As trees age, the branch increment decreases progressively. We employed a multiple linear regression model as the foundational model (Mäkinen, 1999) and gradually introduced variables related to tree characteristics, competition, and climate. Initially, variables with significance levels below 0.05 and the lowest *AIC* values were selected as the first variables for the model. Subsequently, additional variables were tested based on the same criteria starting from the first selected variable and sequentially adding a second variable, a third variable, and so on. Finally, to assess multicollinearity among variables (i.e., excessive correlation between variables), variance inflation factors (VIFs) were employed. Variables with VIF values exceeding 5 were further scrutinized in the selection process.


(5) $$log(\Delta BL)=a_{0}+a_{1}\;*\;AGE+a_{2}\;*\;BL$$

(6) $$log(\Delta BD)=a_{0}+a_{1}\;*\;AGE+a_{2}\;*\;BD$$

In this study, hierarchical partitioning (HP) analysis was employed to assess the relative importance of each variable in the branch growth model (Chevan and Sutherland, 1991). Additionally, the trees were categorized into three different levels, dominant, intermediate, and suppressed, to examine the differential contribution of each variable to branch growth across different tree grades.

### 2.3 Construction of the mixed-effects model

Traditional least squares methods can be used to estimate only the average of the dependent variable, ignoring individual differences in hierarchical data. Mixed-effects models, which include both fixed-effect parameters and random-effect parameters, allow for the analysis of both between-group and within-individual variations in the data. These models have been widely used for the analysis of nested data structures (Fu et al., 2013; Hao et al., 2015). However, considering the practicality of implementing branch-level mixed-effects models in forestry production and the associated sampling costs, we established a linear mixed-effects model for branch growth at the individual tree level. The formula for the model is as follows:


(7) $$\left\{ \begin{array}{c} y_{i}=X_{i}\beta +Z_{i}u_{i}+\epsilon_{ij},\;i=1,\ldots ,n,\\ u_{i}∼N(0,D)\\ \epsilon_{ij}∼N(0,M_{i}) \end{array}\right.$$

where *y_i_* represents the logarithm of branch growth for the $i_{th}$ plot, *i* denotes the known design matrix for fixed effects, *Z_i_* represents the design matrix for random effects, *β* is the vector of fixed-effect parameters, *u_i_* is the vector of random-effect parameters, $\epsilon_{ij}$ signifies the error vector, and *n* denotes the number of sample trees. *D* represents the random-effects covariance matrix. *M_i_* represents the within-group error variance−covariance structure, which describes the heteroscedasticity and autocorrelation present in the data. In forestry, a common form of the *M_i_* matrix is as follows:


(8) $$M_{i}=\sigma^{2}G_{i}^{0.5}\Gamma_{i}G_{i}^{0.5}$$

where *M_i_* represents the error variance−covariance matrix for the $i_{th}$ plot, $\sigma^{2}$ denotes the residual variance of the model, *G_i_* is a diagonal matrix describing within-group heteroscedasticity, and $\Gamma_{i}$ is a matrix accounting for the within-group autocorrelation structure due to multiple measurements on the same tree. Initial analysis suggests the absence of linear heteroscedasticity in the data; thus, *G_i_* is set as a diagonal matrix with all diagonal elements equal to 1. The most commonly employed first-order autoregressive structure (AR (1)) is used to describe the time autocorrelation of the branch growth of individual trees.

### 2.4 Model validation and evaluation

Leave-one-out cross-validation (LOOCV) was employed to evaluate the predictive ability and parameter estimation stability of the branch growth model (Dong et al., 2018). In this study, with a total of 54 individual trees, each dataset was iteratively treated as a validation sample, and the remaining data were used for model building. This process yielded 54 sets of estimated model parameters and corresponding predicted results for branch growth across the 54 individual trees. The following metrics were utilized to assess the model’s goodness of fit and predictive performance: the Akaike information criterion (*AIC*), mean absolute error (*MAE*), mean error (*ME*), mean percentage error (*MPE*), mean absolute percentage error (*MAPE*), and fit index (*FI*). These metrics were utilized to evaluate and compare the predictive performance of the constructed baseline model and the linear mixed-effects model. The specific formulas for calculating these evaluation metrics are as follows:


(9) $$R^{2}=1-\frac{\sum_{i=1}^{n}{\left(y_{i}-{\hat{y}}_{i}\right)}^{2}}{\sum_{i=1}^{n}{\left(y_{i}-{\bar{y}}_{i}\right)}^{2}}$$

(10) $$RMSE=\sqrt{\sum_{i=1}^{n}{\left(y_{i}-{\hat{y}}_{i}\right)}^{2}/(n-p-1)}$$

(11) AIC=−2LL+2p

(12) $$ME=\frac{1}{n}\sum_{i=1}^{n}(y_{i}-{\hat{y}}_{i,-k})$$

(13) $$MAE=\frac{1}{n}\sum_{i=1}^{n}|y_{i}-{\hat{y}}_{i,-k}|$$

(14) $$MPE=\frac{1}{n}\sum_{i=1}^{n}(y_{i}-{\hat{y}}_{i,-k})/\bar{y}$$

(15) $$MAPE=\frac{1}{n}\sum_{i=1}^{n}|y_{i}-{\hat{y}}_{i,-k}|/y_{i}$$

(16) $$FI=1-\frac{\sum_{i=1}^{n}{\left(y_{i}-{\hat{y}}_{i}\right)}^{2}}{\sum_{i=1}^{n}{\left(y_{i}-{\bar{y}}_{i}\right)}^{2}}$$

where *p* represents the number of model parameters; *LL* denotes the log-likelihood value; $y_{i}$ , ${\hat{y}}_{i}$ , and ${\bar{y}}_{i}$ refer to the observed, predicted, and mean values of branch growth, respectively; ${\hat{y}}_{i,-k}$ represents the predicted value of branch growth obtained through the “leave-one-out” approach; and *n* represents the sample size of branches.

## 3 Results

### 3.1 Establishment of the basic model

To construct the base model, we incrementally introduced tree characteristics, competition, and climatic variables by selecting variables that were significant and minimized the Akaike information criterion (*AIC*). There was no multicollinearity among the variables. The parameter estimates and evaluation metrics of the model are presented in **Table 2**, where all the parameter estimates are statistically significant. The parameter estimation results indicate that among the branch-level variables introduced into the model, branch age (*a*_1_) is negatively correlated with branch growth, whereas branch dominance, represented by the cumulative branch length and basal diameter (*a*_2_) and branch height (*a*_3_), is positively correlated with growth. At the tree level, the coefficient estimates for *a*_4_, which are 0.7316 for length and 0.3889 for diameter, represent the mean scaling factors used to model the relationship between branch growth and tree height growth, assuming a statistical allometric relationship. In accounting for climate and competition pressure in the model, the estimates for *a*_5_ and *a*_6_ suggest inverse relationships between branch growth and factors such as age, competition, and climate. Furthermore, *a*_7_, the positive coefficient for the interaction between competition and climate, suggests that the relationships among climate, competition, and branch growth are moderated by their interaction. The final model form was confirmed as follows:

**Table 2:** Basic and mixed-effects parameters, variance components, and model performance of the basic and mixed-effects models of the branch growth models.

Terms	ΔBL model						ΔBD model	
	Basic model		Mixed-effects model		Basic model		Mixed-effects model	
	Estimates	Std	Estimates	Std	Estimates	Std	Estimates	Std
a0	2.894	7.460*10^-2^	1.992	1.263*10^-1^	1.117	1.597*10^-1^	9.657*10^-1^	1.645*10^-1^
a1	−1.111*10^-1^	2.807*10^-3^	−7.064*10^-2^	3.773*10^-3^	−1.321*10^-1^	2.236*10^-3^	−1.098*10^-1^	3.169*10^-3^
a2	4.118*10^-1^	1.206*10^-2^	4.667*10^-1^	1.542*10^-2^	4.023*10^-2^	8.644*10^-4^	3.701*10^-2^	1.054*10^-3^
a3	2.128*10^-2^	1.758*10^-3^	1.707*10^-1^	7.114*10^-3^	1.911*10^-2^	1.518*10^-3^	6.355*10^-2^	5.064*10^-3^
a4	7.316*10^-1^	5.271*10^-2^	8.047*10^-1^	6.106*10^-2^	3.889*10^-1^	4.619*10^-2^	3.002*10^-1^	5.501*10^-2^
a5	−9.738*10^-3^	2.459*10^-3^	−6.997*10^-2^	3.670*10^-3^	−1.355*10^-2^	5.638*10^-3^	−3.743*10^-2^	6.175*10^-3^
a6	−2.369*10^-3^	5.535*10^-4^	−1.985*10^-4^	5.391*10^-4^	−2.043*10^-3^	6.530*10^-4^	−1.871*10^-3^	6.559*10^-4^
a7	5.033*10^-5^	1.981*10^-5^	5.077*10^-5^	1.912*10^-5^	4.806*10^-5^	2.341*10^-5^	5.849*10^-5^	2.332*10^-5^
*Variance and covariance*								
$\sigma^{2}$			0.2018				0.1673	
$\sigma_{u}^{2}$			0.4013				0.0476	
*Fitting statistics*								
*R* ^2^	0.3052		0.3957		0.4446		0.4824	
*RMSE*	8.0353		7.4020		1.0235		0.9884	
*MAE*	6.3101		5.7499		0.7262		0.6981	
*AIC*	8369.815		7945.796		6804.498		6704.655	
*Validation statistics*								
*ME*	1.7667		1.5525		0.1920		0.1835	
*MPE*	8.4825		7.4545		7.6174		7.2806	
*MAE*	6.3817		5.8235		0.7331		0.7039	
*MAPE*	44.5829		40.1349		37.0691		35.4550	
*FI*	0.2909		0.3840		0.4338		0.4741	


(17) $$\begin{array}{c} Log(\Delta BL)=a_{0}+a_{1}*\;AGE+a_{2}*\;BL+a_{3}*\;BH+a_{4}\\ \;*\;Dheight+a_{5}\;*\;BAS+a_{6}\;*\;\\ CMD\_MAM+a_{7}\;*\;BAS\;*\;CMD\_MAM \end{array}$$

(18) $$\begin{array}{c} Log(\Delta BD)=a_{0}+a_{1}\;*\;AGE+a_{2}\;*\;BD+a_{3}\;*\;BH+a_{4}\\ \;*\;Dheight+a_{5}\;*\;BAS+a_{6}\;*\;\\ Eref\_MAM+a_{7}\;*\;BAS\;*\;Eref\_MAM \end{array}$$

To predict the increase in branch length, the model incorporated age (*AGE*), accumulated branch length (*BL*), branch height (*BH*), tree height increment (*Dheight*), basal area per hectare (*BAS*), and spring Hargreaves climatic moisture deficit ( CMD_MAM ). With respect to branch diameter growth, the model included age (*AGE*), accumulated branch diameter (*BD*), branch height (*BH*), tree height increment (*Dheight*), basal area per hectare (*BAS*), and spring Hargreaves relative evapotranspiration ( Eref_MAM ). Parameters *a*_0_, *a*_1_, *a*_2_, *a*_3_, *a*_4_, *a*_5_, *a*_6_, and *a*_7_ represent model parameters. The model’s parameter estimates and evaluation metrics are detailed in **Table 2**, where all the parameter estimates are statistically significant.

### 3.2 Establishment of the mixed-effects model

We attempted to incorporate random effects at the plot level into intercept terms and each predictor variable, determining their optimal positions based on *AIC* values and likelihood ratio tests (LRTs). The final optimal mixed-effects model was identified as follows:


(19) $$\begin{array}{c} Log(\Delta BL)=a_{0}+a_{1}\;*AGE+a_{2}\;*\;BL+a_{3}\;*\;BH\\ +a_{4}\;*\;Dheight+a_{5}\;*\;BAS+a_{6}\;*\;\\ CMD\_MAM+a_{7}\;*\;BAS\;*\;CMD\_MAM+u_{0} \end{array}$$

(20) $$\begin{array}{c} Log(\Delta BD)=a_{0}+a_{1}\;*\;AGE+a_{2}\;*\;BD+a_{3}\;*\;BH\\ +a_{4}\;*\;Dheight+a_{5}\;*\;BAS+a_{6}\;*\;\\ Eref\_MAM+a_{7}\;*\;BAS\;*\;Eref\_MAM+u_{1} \end{array}$$

where *u*_0_ and *u*_1_ represent the random effects for branch length and diameter growth, respectively, with other symbols defined earlier.

### 3.3 Model evaluation and validation

**Figure 2:**
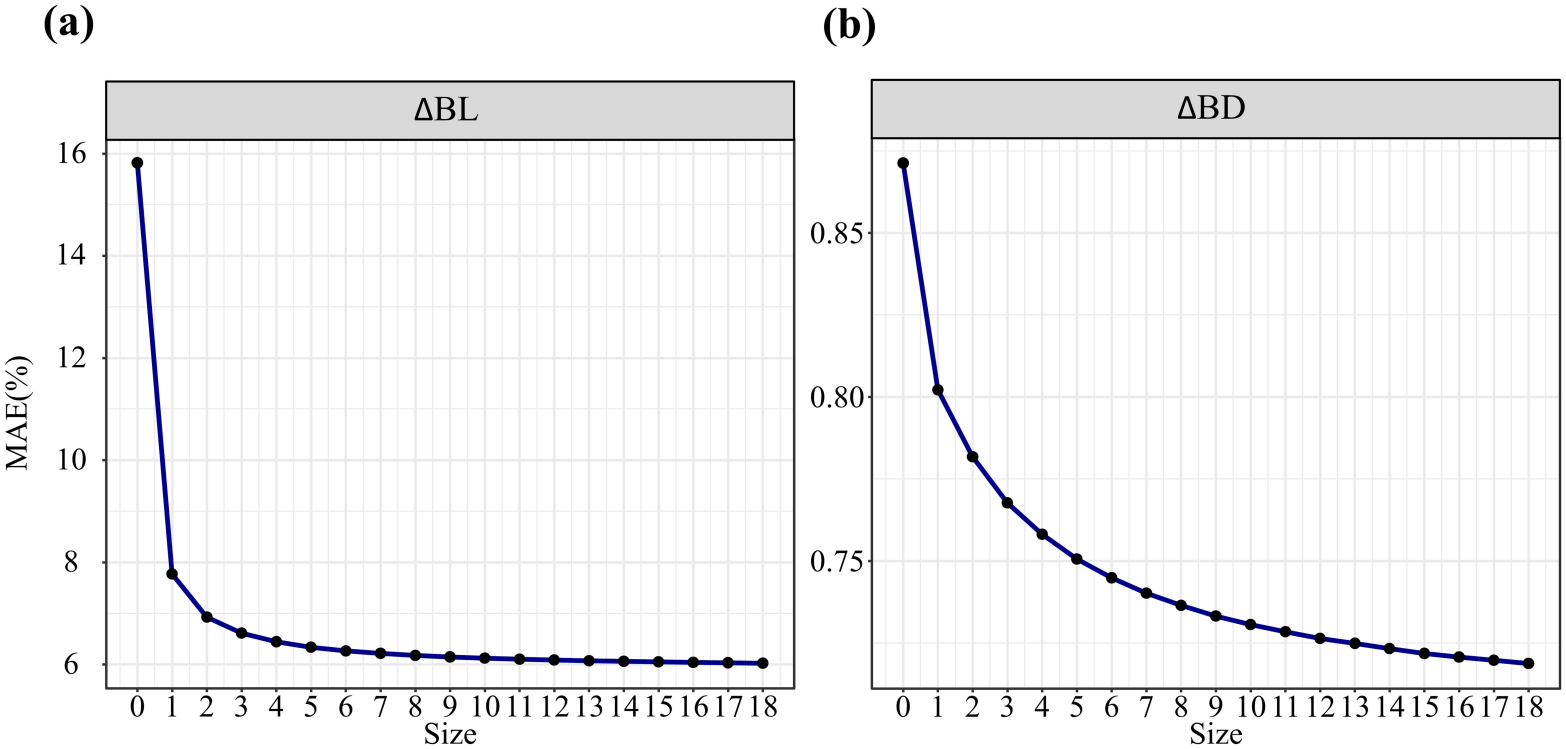
MAE with different subsampled branch sizes for the mixed-effects model and the fixed model (sample size = 0): calibration for the annual growth of branch length **(A)** and branch diameter **(B)**.

**Table 2** presents the parameter estimates, fit statistics, and leave-one-out cross-validation statistics of the model. Compared with the base model, the mixed-effects model significantly improved various fit indices; for example, the R-squared value for branch length growth increased by 0.09, and that for branch diameter growth increased by 0.05. Comparative metrics indicate that the mean prediction error (*MPE*) and mean absolute error (*MAE*) decreased by 1.03 and 0.56, respectively, for branch length growth and by 0.42 and 0.03, respectively, for branch diameter growth, suggesting that the mixed-effects model provides better predictive performance in forecasting branch growth based on prior information. We further analyzed the potential impact of sample size on model predictions. As depicted in **Figure 2**, regardless of the sample size, the calibrated prediction accuracy significantly surpassed the uncalibrated prediction accuracy (sample size = 0). With increasing sample size, the accuracy of the mixed-effects models consistently improved. The variation in prediction accuracy stabilized when the number of branches reached a value of approximately six. The variation in the prediction accuracy of the branch length growth models stabilized when there were approximately 2–3 branches, whereas for the branch diameter growth models, this stabilization occurred at approximately 6 branches.

### 3.4 Factors influencing branch growth

**Figure 3:**
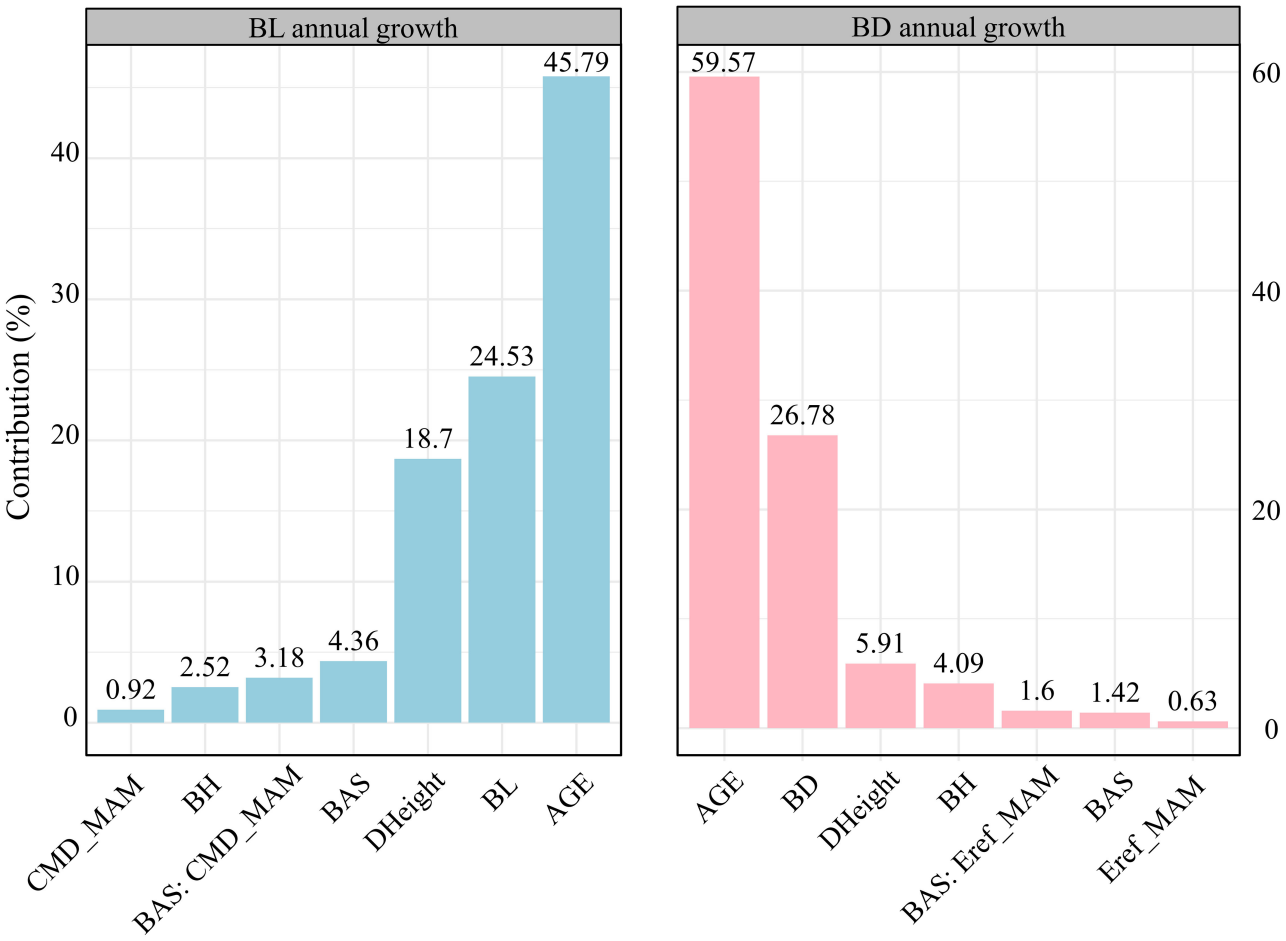
Bar plots of the relative importance of variables such as branch age, branch size, branch height, tree height growth, competition, climate, and their interactions on annual growth in terms of branch length (left) and branch diameter (right) via the hierarchical partitioning method. Specific abbreviations are detailed in **Table 1**.

**Figure 4:**
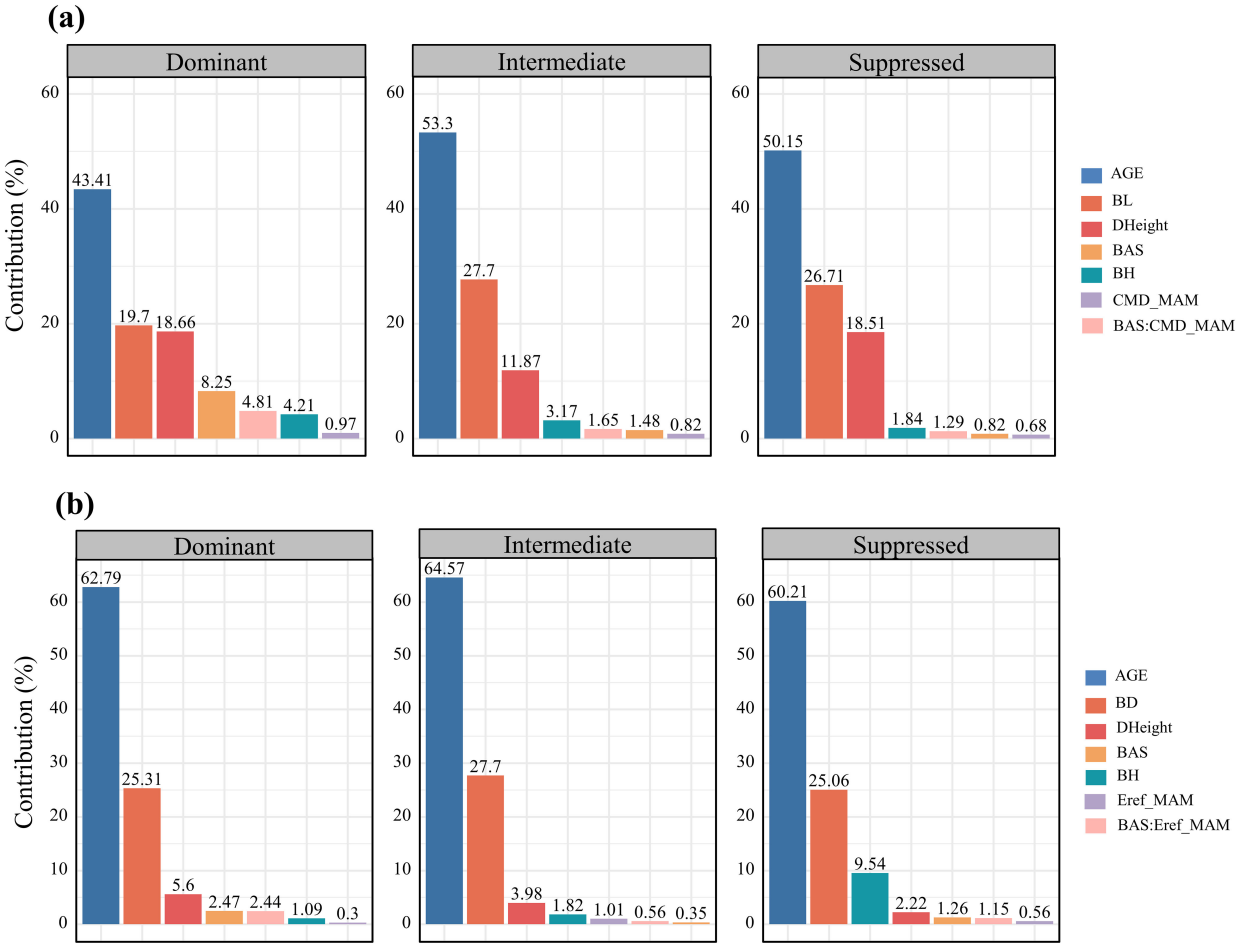
Bar plots of the relative importance of variables in annual growth across trees with different social statuses in terms of branch length **(A)** and branch diameter **(B)** trees in HP analysis. The variables in the doughnut chart are explained above.

Hierarchical analysis revealed that branch age was the most significant factor influencing both branch length and diameter growth (over 45%), followed by branch size (24.53% for length and 26.78% for diameter) (**Figure 3**). The increase in tree height ranked third, affecting primarily branch length growth (18.7%). In terms of responses to climatic and competitive variables, differences emerged in the growth of branch length and diameter. For branch length growth, competitive effects (4.36%) > interactive effects between climate and competition (3.18%) > climate effects alone (0.92%). Conversely, for branch diameter growth, the interactive effects between climate and competition (1.6%) > competitive effects (1.42%) > climate effects alone (0.63%). **Figure 4** illustrates the variations in the contributions of variables across trees with different social statuses (dominant trees, intermediate trees, and suppressed trees). Branch length growth, branch height, competition, climate, and their interactions had the strongest correlations with branch growth in the dominant trees. For branch diameter growth, branch height had a relatively greater influence on suppressed trees; competition and the interaction between competition and climate had the greatest influence on dominant trees; and climate had the greatest correlation with growth among intermediate trees. Overall, in the dominant trees, the independent effects of climate and competition, as well as the interactive effects between them, appeared more pronounced than those in the intermediate and suppressed trees.

### 3.5 Response of branch growth to variables

**Figure 5:**
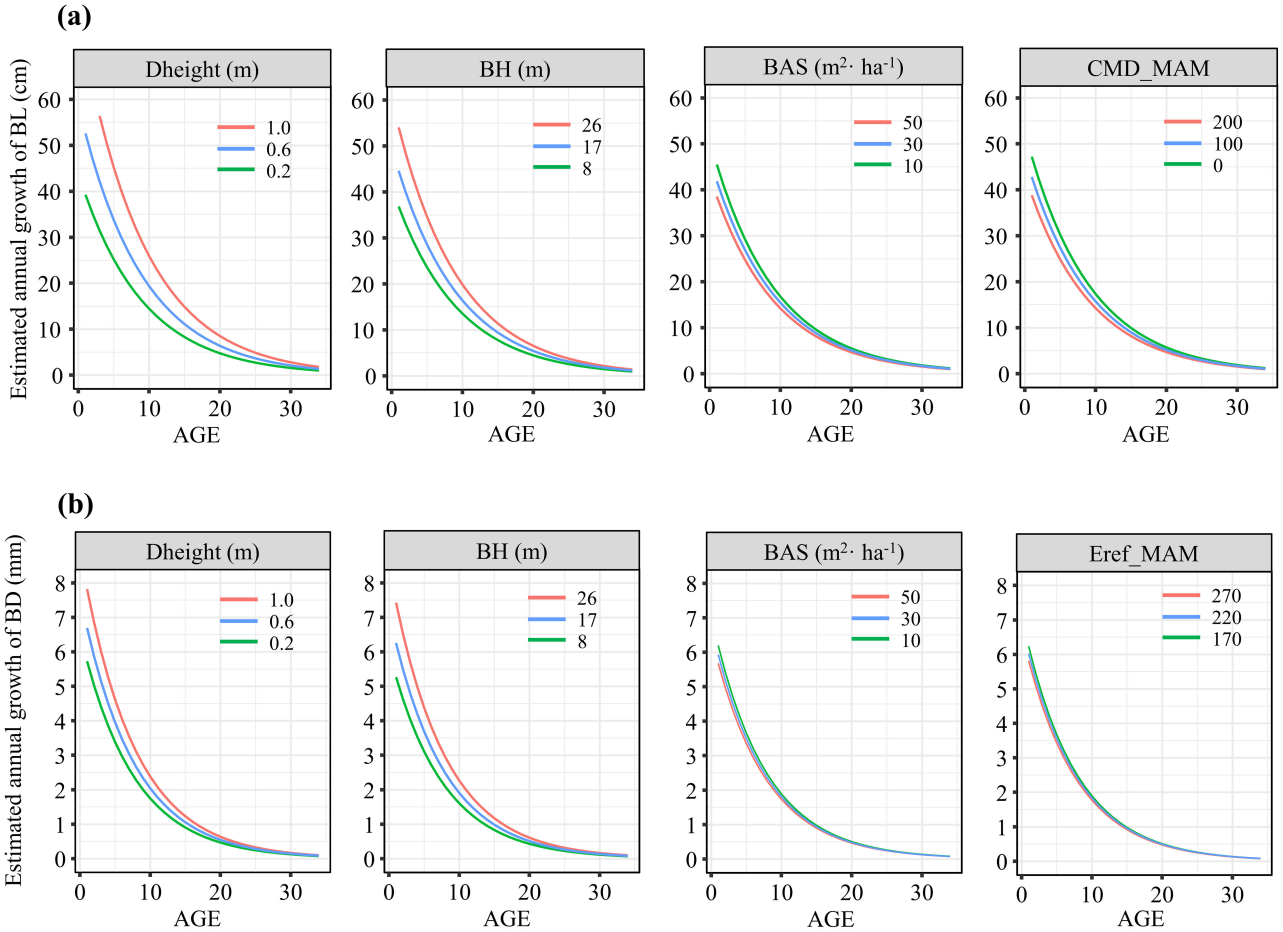
Effects of gradients of different variables on the annual growth of branch length **(A)** and branch diameter **(B)**.

**Figure 6:**
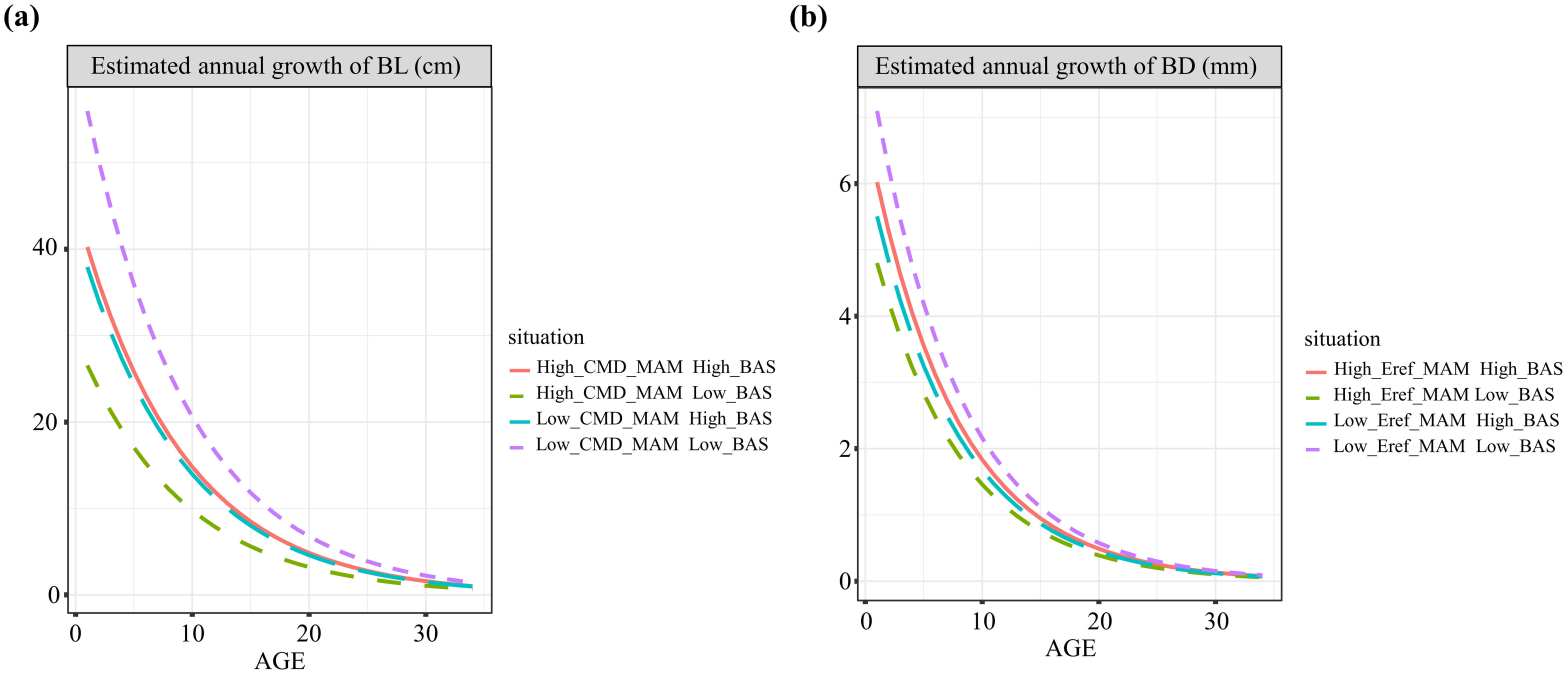
Age-based annual growth curves of branch length **(A)** and branch diameter **(B)** with different combinations of competitive and climatic variables.

To better illustrate the independent impacts of various variables on branch growth, the relationships between branch growth and branch age across three gradients of variables (equally spaced ranges) and average values of other variables are depicted in **Figure 5**. Both tree height increment and branch height were positively correlated with branch length and diameter growth. However, as the sum of the basal area per hectare and climatic variables increased, both branch length and diameter growth tended to decrease. Additionally, we examined how the interactive effects between competition and climate varied across different pressure gradients (**Figure 6**). For both branch length and diameter growth, the maximum growth occurred only when both climate and competition pressures were low. When either climate or competition pressures were high, branch growth was significantly reduced, with minimal distinction between the effects of climate and competition. When climate stress was elevated, the intensity of competition had a pronounced effect on the variation in branch growth.

## 4 Discussion

### 4.1 Development of branch growth models

Branches provide fundamental information on the developmental patterns of tree crowns over short periods (Osada, 2011). Branch growth models can bridge the gap between traits studied extensively at the leaf level (e.g., photosynthesis) and the crown level (e.g., allometric growth) (Osada, 2006). Recently, constructing biologically meaningful branch growth models based on easily measurable stand- and tree-level variables has emerged as a convenient and reliable approach to determine tree growth dynamics (Dong et al., 2015; Zou et al., 2024). These static models often assume that branches of all sizes within a whorl are equally influenced by stand and tree factors, which may not fully capture developmental realities. In this study, we employed dynamic variables to reconstruct the growth patterns of branches across different ages, aiming to link branch traits with various limiting factors. Mixed-effects models are commonly used to address hierarchical data structures effectively (Meteyard and Davies, 2020). Our results show that linear mixed-effects models can be used to predict data accurately from independent datasets, with improved precision compared with fixed-effects models. To mitigate the challenges of measuring all branches within a crown during model calibration, we investigated the impact of the number of sampled branches per tree on model predictive performance. Our findings suggest that increasing the sample size enhances model accuracy. Notably, when the sample size for branches is 2–3, the steep curve of the branch length growth model stabilizes, indicating that selecting 2–3 branches for predicting branch length growth is a cost-effective calibration strategy in practical applications. Moreover, the predictive accuracy of branch diameter growth consistently improves as the sample size increases, with only marginal improvements in accuracy (less than 0.5%) when the sample size exceeds six. Therefore, we conclude that the measurement of 6 branches is the most cost-effective sampling strategy, as it achieves a balance between model performance and economic cost.

### 4.2 Impact of variables on branch growth

Few studies have integrated tree height growth patterns with branch growth models. Research on vegetation height growth and biomass allocation in tropical rainforests suggests that the optimal strategies for maintaining tree height growth balance involve “horizontal crown expansion for maximum light acquisition” and “increased biomass allocation to height growth to improve light conditions” (Matsuo et al., 2024). Furthermore, correlations between branch growth patterns, branch quantity and tree height have been established (Weiskittel et al., 2007). Thus, in our model, dynamic tree height growth is introduced as a key variable that significantly explains the influence of stand conditions on branch growth variability. The results revealed a positive relationship between branch growth and tree height growth, possibly because tree vigor is associated with tree height growth (Remphrey and Powell, 1984; Mäkinen, 1999; Mäkinen and Colin, 1999). Trees tend to allocate more resources (e.g., nitrogen) to branches that thrive under optimal crown environments (Chen and Sumida, 2018). Consequently, resource exploitation competition between shaded and sunlit branches within the same crown can affect branch growth. Therefore, branch height, which reflects branch position within the crown, is crucial to consider in the model. We observed that branch height and branch growth increased positively with branch age. From a physiological perspective, the auxins that regulate various developmental processes in plants are predominantly located in regions of active growth, whereas their distribution is minimal in aging organs and tissues (Khan et al., 2024). Additionally, as a species with prominent apical dominance, the growth of lateral branches in red pine is regulated by the main stem, resulting in varying growth rates of the lateral branches from top to bottom (Kebrom, 2017). This can be attributed to the fact that taller branches receive more light, promoting growth, whereas lower-crown branches face intense resource competition, limiting growth (Chen and Sumida, 2017). Previous studies have also confirmed that the hydraulic conductivity efficiency of high branches is not reduced due to height and gravity effects; instead, these branches can store more water for on-demand retrieval (Burgess et al., 2006).

Competition has been demonstrated to significantly correlate with branch growth, survival, crown structure, and functionality in terms of nutrient transport and crown resource allocation (Labyak and Schumacher, 1954; Sprugel, 2002; Weiskittel et al., 2007). Unlike previous studies that focused on competitive variables in branch growth research, this study reconstructs a retrospective dynamic competition index as an individual-based approach, facilitating a clear understanding of competition variations over time and space. We primarily consider commonly used distance-dependent and distance-independent dynamic competition indices, where the former reflects individual competitive ability (Lorimer, 1983) and the latter quantifies competition among individuals through the available growth space (Smith and Bell, 1983). The results indicate that the distance-independent competition index—the sum of stand basal area—yields better model efficacy, suggesting a stronger correlation between stand basal area and branch growth. This aligns with previous findings suggesting the applicability and universality of distance-independent competition indices in managed forests (Prévosto et al., 2000; Guo et al., 2023). The stand basal area is a critical measure of stand density that is widely applied in forest planning and management (Zhao et al., 2020). Compared with other competition indices, it is easier to acquire and computationally straightforward, justifying its inclusion in branch growth models. The negative relationship between competition and branch growth indicates a causal effect: increased stand density can limit growth space for individual trees, thereby constraining crown expansion. Studies also confirm that denser stands cause smaller crown sizes (Mäkinen and Hein, 2006; Benomar et al., 2012). Moreover, an increase in stand density intensifies competition for light, water, and nutrients, potentially restricting branch growth (Primicia et al., 2014).

Our study confirms the significant effects of seasonal climatic variables on branch growth. Overall, branch growth in the first two years is correlated primarily with reference evapotranspiration (*Eref*) and climatic moisture deficit (*CMD*) in spring, which are identified as the most significant factors through stepwise regression analysis. These two variables not only provide multidimensional climatic information from the perspective of water and energy cycles but also reveal how climate factors (such as precipitation and temperature changes) affect branch growth and development through the evapotranspiration process (Abtew and Melesse, 2013). These findings further support the role of *Eref* and *CMD* as core indicators in studying the impact of climate change on branch growth. One of the harmful effects plants commonly face is water scarcity (Haghpanah et al., 2024). Preseason moisture plays an increasingly important role in tree growth and wood formation during the growing season (Li et al., 2022). Specifically, *Eref* and *CMD* in spring jointly determine the availability of water during both spring and the growing season. The increases in evaporation, transpiration, and moisture loss can result in rapid water depletion in spring, which affects branch development in various ways, including reduced cell expansion, carbon supply, and increased susceptibility to pests and pathogens (Caldeira, 2019; Stephenson et al., 2019). Higher *Eref* and *CMD* values increase the likelihood of drought and water stress during the subsequent growth season. This leads to a decline in photosynthesis, a reduction in growth-promoting hormones, and an increase in growth-inhibiting hormones, all of which suppress branch and tree growth (Tezara et al., 1999).

Climate and competition have been focal points in many studies on branch growth, yet their interaction has received little attention. For the first time, we introduce the interaction between climate and competition into studies of branch growth and confirm the significant effects of these interactions on branch growth and the increased predictive accuracy of models that account for them. Studies on hardwoods have demonstrated that the interaction between competition and climate has a greater impact on tree growth than does climate alone, highlighting the critical regulatory role of competition in tree growth responses to climate (D’Amato et al., 2013; Ford et al., 2017). Consistent with these findings, our results demonstrate that both climate and competition negatively influence branch growth, whereas their interaction yields a positive effect. We suggest that this phenomenon may be attributed to resource limitations and growth optimization mechanisms. Under typical conditions, competition and environmental stress exacerbate resource scarcity, thereby inhibiting branch growth. However, the interaction between climate and competition may, under specific circumstances, increase branch growth by modulating resource allocation and utilization strategies (Wang et al., 2022a). Additionally, we present the variations in branch growth under different combinations of competition and climatic conditions. Trees exhibit differential responses to environmental factors, as they adapt their growth strategies to optimize performance under varying levels of competition and climatic stress (Albert and Schmidt, 2010). In general, under conditions of low competition and minimal climatic stress, branches are able to fully exploit their growth potential, as resources are not constrained by intense competition, leading to more vigorous growth (Carnwath et al., 2012). In contrast, under high competition or significant climatic stress, environmental factors may limit branch growth; however, when competition pressure is relatively low, climatic conditions can positively influence branch growth, with the interaction between these two factors manifesting a beneficial effect in such contexts. Therefore, we predict that the impact of climate change on branch growth is likely to increase growth under relatively low-stress (i.e., adequate energy supply) conditions and is maximal in stands with relatively low competition. Furthermore, our findings suggest that optimal climates typically support better branch growth but may intensify resource competition, amplifying growth disparities among branches of different sizes. Overall, our study underscores the intricate interplay between climate and competition in shaping branch growth dynamics, highlighting the need for integrated approaches in understanding forest ecosystem responses to environmental changes (Blake et al., 1979; Yan et al., 2023).

### 4.3 Impact of competition and climate mediated by social status

The results suggest that tree social status significantly influences the sensitivity of branches to various variables. Trees of different social statuses acquire resources in different ways through various physiological processes. Thus, competition and climate within stands can have differential effects on the branch growth of trees categorized as individuals with different social statuses. Specifically, for dominant trees, the contributions of competition, climate, and their interaction are greater than those for intermediate and suppressed trees. This suggests that dominant trees are more sensitive to symmetric competition driven by stand density and to growth inhibition or promotion caused by climatic constraints. In contrast, the growth of branches in intermediate and suppressed trees is more influenced by internal competitive dynamics, such as the competitive status of the branches within the crown (reflected in age and cumulative size) and the vitality of the trees under competitive pressure (reflected in height growth). However, the independent effect of climate appears to have the least impact on suppressed trees (Yan et al., 2023), with less impact from competition. Dominant and intermediate-sized individuals, which are in more active growth phases, require more resources, increasing their susceptibility to competitive pressures (Arzac et al., 2021). The varying responses of individuals at different social statuses to climate may reflect differences in microclimatic conditions and differences in light and resource availability. Suppressed individuals experience less radiation, lower vapor pressure deficits, and less storm impact, fostering microclimates that buffer these trees against climatic stresses. Conversely, dominant and intermediate-sized individuals, which are more exposed to direct climate environments, may exhibit greater sensitivity to climate variability (Mérian and Lebourgeois, 2011; Wang et al., 2022a; Yang et al., 2022). Furthermore, studies suggest that suppressed individuals have shorter growing seasons and delayed growth initiation, which reduces their exposure to extreme climatic events, making them less vulnerable to climate-induced stress. These findings underscore the differential impacts of competition and climate on branch growth across trees with different social statuses, underscoring the complexity of forest dynamics in response to environmental factors (Liu et al., 2018).

### 4.4 Insights for adaptive management practices

As global climate issues intensify and the area of natural forests rapidly declines, plantations are playing an increasingly critical role in addressing climate change and ecological conservation (Roy, 2024). Plantations are designed to provide various ecosystem services, primarily timber and other wood products (Pawson et al., 2013). However, while focusing on timber yield, i.e., tree growth, it is also important to consider the balance between the benefits (such as carbon sequestration, oxygen release, and providing habitats for wildlife) and drawbacks (such as reduced wood quality) associated with branch growth (Brockerhoff et al., 2017; Kellomäki, 2022). In this study, we analyzed the correlations between tree growth and branch growth, as well as the factors that impact these variables, such as climate and competition, and revealed that trees of different statuses respond differently to environmental factors. Therefore, to increase the resilience and adaptability of forests to climate change, we propose the following recommendations for future forest management. First, the demonstrated importance of the interaction between competition and climate in branch growth models reinforces the need to focus on this interaction in future models to increase model reliability. This interaction should be better understood and integrated into models to improve their predictive capacity. Second, the greater branch growth observed under low climatic stress and low competition levels suggests that stand competition should be adjusted to mitigate climate stress. Adjusting competition appropriately (such as by increasing the planting density or implementing selective thinning measures) based on climate conditions and management objectives can improve branch growth. Finally, our study highlights that the sensitivity of branch growth to competition and climate is mediated by tree social status. Suppressed individuals exhibit lower sensitivity to competition and climate. Therefore, in future research into the dynamics of branch growth in relation to climate–competition sensitivity, selective sampling based on tree social status should be considered to obtain more valuable insights. These insights provide guidance for implementing adaptive forestry management practices that consider the complex interplay between competition, climate, and tree social status to optimize branch growth and enhance forest resilience in the face of changing environmental conditions.

## 5 Conclusions

This study provides the first comprehensive analysis of how tree variables, climate, competition, and their interactions affect branch growth dynamics. We emphasize the importance of not overlooking the interaction between competition and climate, as its relative significance can even surpass that of the individual effects of climate. Moisture stress emerges as a key variable influencing branch growth, with its effects regulated by competition intensity (stand basal area). Additionally, the differences in variable importance resulting from varying tree social statuses suggest that sampling dominant and intermediate-sized individuals may provide insights into the relationships among competition, climate, and branch growth. Although our study represents limited-scale research based on a specific tree species and forest stand type, the findings offer useful insights for forest management. Although direct applicability to other species or stand types requires caution, the identified interactions, especially those between competition and climate, are likely relevant across diverse forest ecosystems. The focus on moisture stress and competition intensity lays a foundation for adaptive management strategies in varying forest conditions. This study provides management recommendations for addressing climate stress on branch growth, aiding both short- and long-term forest management goals, and offering new directions for climate change adaptation.

## Data Availability

The raw data supporting the conclusions of this article will be made available by the authors, without undue reservation.

## References

[B1] Abtew W. Melesse A. (2013). “Climate change and evapotranspiration,” in Evaporation and Evapotranspiration: Measurements and Estimations. Eds. Abtew W. Melesse A. (Springer Netherlands, Dordrecht), 197–202.

[B2] Administration, N.F.a.G (2019). China Forest Resources Report 2014-2018. Ed. Zhang J. (Beijing: China Forestry Publishing House).

[B3] Albert M. Schmidt M. (2010). Climate-sensitive modelling of site-productivity relationships for Norway spruce (Picea abies (L.) Karst.) and common beech (Fagus sylvatica L.). For. Ecol. Manage. 259, 739–749. doi: 10.1016/j.foreco.2009.04.039

[B4] Arzac A. Tabakova M. A. Khotcinskaia K. Koteneva A. Kirdyanov A. V. Olano J. M. (2021). Linking tree growth and intra-annual density fluctuations to climate in suppressed and dominant Pinus sylvestris L. trees in the forest-steppe of Southern Siberia. Dendrochronologia 67, 125842. doi: 10.1016/j.dendro.2021.125842

[B5] Benomar L. Benomar L. DesRochers A. Larocque G. R. (2012). The effects of spacing on growth, morphology and biomass production and allocation in two hybrid poplar clones growing in the boreal region of Canada. Trees. 26, 939–949. doi: 10.1007/s00468-011-0671-6

[B6] Blake J. Zaerr J. Hee S. (1979). Controlled moisture stress to improve cold hardiness and morphology of douglas-fir seedlings. For. Sci. 25, 576–582. doi: 10.1093/forestscience/25.4.576

[B7] Brockerhoff E. G. Barbaro L. Castagneyrol B. Forrester D. I. Gardiner B. González-Olabarria J. R.. (2017). Forest biodiversity, ecosystem functioning and the provision of ecosystem services. Biodiversity Conserv. 26, 3005–3035. doi: 10.1007/s10531-017-1453-2

[B8] Burgess S. S. O. Pittermann J. Dawson T. E. (2006). Hydraulic efficiency and safety of branch xylem increases with height in Sequoia sempervirens (D. Don) crowns. Plant Cell Environ. 29, 229–239. doi: 10.1111/j.1365-3040.2005.01415.x 17080638

[B9] Caldeira M. C. (2019). The timing of drought coupled with pathogens may boost tree mortality. Tree Physiol. 39, 1–5. doi: 10.1093/treephys/tpy141 30615167

[B10] Carnwath G. C. Peterson D. W. Nelson C. R. (2012). Effect of crown class and habitat type on climate–growth relationships of ponderosa pine and Douglas-fir. For. Ecol. Manage. 285, 44–52. doi: 10.1016/j.foreco.2012.07.037

[B11] Chen L. Sumida A. (2017). Patterns of branch growth and death in crowns of sakhalin spruce, picea glehnii (F. Schmidt) mast. Forests 8, 26. doi: 10.3390/f8010026

[B12] Chen L. Sumida A. (2018). Effects of light on branch growth and death vary at different organization levels of branching units in Sakhalin spruce. Trees 32, 1123–1134. doi: 10.1007/s00468-018-1700-5

[B13] Chen X. Willis J. L. Bowman K. A. (2022). Assessing the influence of climate on cone production of longleaf pine forests. Trees Forests People 9, 100297. doi: 10.1016/j.tfp.2022.100297

[B14] Chevan A. Sutherland M. (1991). Hierarchical partitioning. Am. Statistician 45, 90–96. doi: 10.1080/00031305.1991.10475776

[B15] Cline M. G. (1997). Concepts and terminology of apical dominance. Am. J. Bot. 84, 1064. doi: 10.2307/2446149 21708661

[B16] Cline M. G. Bhave N. Harrington C. A. (2009). The possible roles of nutrient deprivation and auxin repression in apical control. Trees 23, 489–500. doi: 10.1007/s00468-008-0294-8

[B17] D’Amato A. W. Bradford J. B. Fraver S. Palik B. J. (2013). Effects of thinning on drought vulnerability and climate response in north temperate forest ecosystems. Ecol. Appl. 23, 1735–1742. doi: 10.1890/13-0677.1 24555305

[B18] Deleuze C. Hervé J.-C. Colin F. Ribeyrolles L. (1996). Modelling crown shape of Piceaabies: spacing effects. Can. J. For. Res. 26, 1957–1966. doi: 10.1139/x26-221

[B19] de Reffye P. Kang M. Hua J. Auclair D. (2012). Stochastic modelling of tree annual shoot dynamics. Ann. For. Sci. 69, 153–165. doi: 10.1007/s13595-011-0151-6

[B20] Dong L. Liu Z. Li F. Jiang L. (2015). Modelling primary branch growth based on a multilevel nonlinear mixed−effects model: a Pinus koraiensis plantation case study in north−east China. South. Forests: J. For. Sci. 77, 179–190. doi: 10.2989/20702620.2014.1001676

[B21] Dong L. Zhang L. Li F. (2018). Additive Biomass Equations Based on Different Dendrometric Variables for Two Dominant Species (Larix gmelini Rupr. and Betula platyphylla Suk.) in Natural Forests in the Eastern Daxing’an Mountains, Northeast China. Forests 9, 261. doi: 10.3390/f9050261

[B22] Ford K. R. Breckheimer I. K. Franklin J. F. Freund J. A. Kroiss S. J. Larson A. J.. (2017). Competition alters tree growth responses to climate at individual and stand scales. Can. J. For. Res. 47, 53–62. doi: 10.1139/cjfr-2016-0188

[B23] Fu L. Sun H. Sharma R. P. Lei Y. Zhang H. Tang S. (2013). Nonlinear mixed-effects crown width models for individual trees of Chinese fir (Cunninghamia lanceolata) in south-central China. For. Ecol. Manage. 302, 210–220. doi: 10.1016/j.foreco.2013.03.036

[B24] Gao H. Liu Q. Song Y. Jiang M. Yin Y. (2022). Modeling primary branch diameter and length for planted pinus koraiensis by incorporating neighbor competition in Northeast China. Forests 13, 912. doi: 10.3390/f13060912

[B25] Gleason K. E. Bradford J. B. Bottero A. D’Amato A. W. Fraver S. Palik B. J.. (2017). Competition amplifies drought stress in forests across broad climatic and compositional gradients. Ecosphere 8, e01849. doi: 10.1002/ecs2.1849

[B26] Gleason S. M. Stephens A. E. A. Tozer W. C. Blackman C. J. Butler D. W. Chang Y.. (2018). Shoot growth of woody trees and shrubs is predicted by maximum plant height and associated traits. Funct. Ecol. 32, 247–259. doi: 10.1111/1365-2435.12972

[B27] Guo H. Jia W. Li D. Sun Y. Wang F. Zhang X. (2023). Modelling branch growth of Korean pine plantations based on stand conditions and climatic factors. For. Ecol. Manage. 546, 121318. doi: 10.1016/j.foreco.2023.121318

[B28] Haghpanah M. Hashemipetroudi S. Arzani A. Araniti F. (2024). Drought tolerance in plants: physiological and molecular responses. Plants 13, 2962. doi: 10.3390/plants13212962 39519881 PMC11548289

[B29] Hao X. Yujun S. Wang X. Jin W. Yao F. (2015). Linear mixed-effects models to describe individual tree crown width for China-fir in Fujian Province, Southeast China. PloS One 10, e0122257. doi: 10.1371/journal.pone.0122257 25876178 PMC4398382

[B30] Hegyi F. (1974). A simulation model for managing jack-pine stands simulation. RoyalColl. For, Res. Notes 30, 74–90.

[B31] Hein S. (2008). Knot attributes and occlusion of naturally pruned branches of Fagus sylvatica. For. Ecol. Manage. 256, 2046–2057. doi: 10.1016/j.foreco.2008.07.033

[B32] Helluy M. Prévosto B. Cailleret M. Fernandez C. Balandier P. (2020). Competition and water stress indices as predictors of Pinus halepensis Mill. radial growth under drought. For. Ecol. Manage. 460, 117877. doi: 10.1016/j.foreco.2020.117877

[B33] Hu M. Lehtonen A. Minunno F. Mäkelä A. (2020). Age effect on tree structure and biomass allocation in Scots pine (Pinus sylvestris L.) and Norway spruce (Picea abies [L.] Karst.). Ann. For. Sci. 77, 90. doi: 10.1007/s13595-020-00988-4

[B34] Ishii H. Clement J. P. Shaw D. C. (2000). Branch growth and crown form in old coastal Douglas-fir. For. Ecol. Manage. 131, 81–91. doi: 10.1016/S0378-1127(99)00202-9

[B35] Jiang Y. Wang Z. Chen H. Hu Y. Qu Y. Chhin S.. (2023). A Bayesian network model to disentangle the effects of stand and climate factors on tree mortality of Chinese fir plantations. Front. Forests Global Change 6. doi: 10.3389/ffgc.2023.1298968

[B36] Jin X. Pukkala T. Li F. Dong L. (2017). Optimal management of Korean pine plantations in multifunctional forestry. J. Forestry Res. 28, 1027–1037. doi: 10.1007/s11676-017-0397-4

[B37] Kaitaniemi P. Lintunen A. Sievänen R. (2020). Power-law estimation of branch growth. Ecol. Model. 416, 108900. doi: 10.1016/j.ecolmodel.2019.108900

[B38] Kanazawa Y. Ishii H. T. Homma S. Kita Y. Jomura M. Wenjie W.. (2013). Comparison of branch growth estimates in Larix gmelinii by several methods. J. For. Res. 18, 345–352. doi: 10.1007/s10310-012-0347-0

[B39] Kebrom T. H. (2017). A growing stem inhibits bud outgrowth – the overlooked theory of apical dominance. Front. Plant Sci. 8. doi: 10.3389/fpls.2017.01874 PMC5671643 29163599

[B40] Kellomäki S. (2022). “Pruning of branchesBranches and management of timber quality,” in Management of Boreal Forests: Theories and Applications for Ecosystem Services. Ed. Kellomäki S. (Springer International Publishing, Cham), 465–476.

[B41] Khan S. Alvi A. F. Khan N. A. (2024). Role of ethylene in the regulation of plant developmental processes. Stresses 4, 28–53. doi: 10.3390/stresses4010003

[B42] Khansaritoreh E. Dulamsuren C. Klinge M. Ariunbaatar T. Bat-Enerel B. Batsaikhan G.. (2017). Higher climate warming sensitivity of Siberian larch in small than large forest islands in the fragmented Mongolian forest steppe. Global Change Biol. 23, 3675–3689. doi: 10.1111/gcb.13750 28470864

[B43] Kuehne C. Weiskittel A. R. Waskiewicz J. (2019). Comparing performance of contrasting distance-independent and distance-dependent competition metrics in predicting individual tree diameter increment and survival within structurally-heterogeneous, mixed-species forests of Northeastern United States. For. Ecol. Manage. 433, 205–216. doi: 10.1016/j.foreco.2018.11.002

[B44] Labyak L. F. Schumacher F. X. (1954). The contribution of its branches to the main-stem growth of loblolly pine. J. Forestry 52, 333–337. doi: 10.1093/jof/52.5.333

[B45] Li Y. Pan Y. Li X. Zhao J. Shi F. Wu X.. (2022). Legacy effect of extreme wetness events on subsequent tree growth evidenced by water use source shifts in a semi-arid region of North China. Trees 36, 967–976. doi: 10.1007/s00468-021-02263-z

[B46] Li X. Zhao M. Xu Y. Li Y. Tigabu M. Zhao X. (2021). Genetic diversity and population differentiation of pinus koraiensis in China. Horticulturae 7, 104. doi: 10.3390/horticulturae7050104

[B47] Liang R. Sun Y. Qiu S. Wang B. Xie Y. (2023). Relative effects of climate, stand environment and tree characteristics on annual tree growth in subtropical Cunninghamia lanceolata forests. Agric. For. Meteorology 342, 109711. doi: 10.1016/j.agrformet.2023.109711

[B48] Liu J. Feng J. Gao H. Chen D. (2024). Nonlinear mixed-effect branch growth model development for planted Korean pine in Northeast China. Trees 38, 409–421. doi: 10.1007/s00468-024-02490-0

[B49] Liu S. Li X. Rossi S. Wang L. Li W. Liang E.. (2018). Differences in xylogenesis between dominant and suppressed trees. Am. J. Bot. 105, 950–956. doi: 10.1002/ajb2.1089 29874391

[B50] Liu S. Liu Y. Wu L. Yi X. Sun H. (2023b). Examining the interactive effects of neighborhood characteristics and environmental conditions on height-to-diameter ratio of Chinese fir based on random forest. For. Ecol. Manage. 544, 121189. doi: 10.1016/j.foreco.2023.121189

[B51] Liu K.-L. Wang C.-S. Chen B.-Y. Wang R.-H. Zeng J. (2023a). Branch development in monoculture and mixed-species plantations of Betula alnoides, Erythrophleum fordii and Pinus kesiya var. langbianensis in southwestern China. For. Ecol. Manage. 528, 120643. doi: 10.1016/j.foreco.2022.120643

[B52] Lorimer C. G. (1983). Tests of age-independent competition indices for individual trees in natural hardwood stands. For. Ecol. Manage. 6, 343–360. doi: 10.1016/0378-1127(83)90042-7

[B53] Madrigal-González J. Andivia E. Zavala M. A. Stoffel M. Calatayud J. Sánchez-Salguero R.. (2018). Disentangling the relative role of climate change on tree growth in an extreme Mediterranean environment. Sci. Total Environ. 642, 619–628. doi: 10.1016/j.scitotenv.2018.06.064 29909329

[B54] Mäkinen H. (1999). Effect of stand density on radial growth of branches of Scots pine in southern and central Finland. Can. J. For. Res. 29, 1216–1224. doi: 10.1139/x99-060

[B55] Mäkinen H. (2002). Effect of stand density on the branch development of silver birch (Betula pendula Roth) in central Finland. Trees 16, 346–353. doi: 10.1007/s00468-002-0162-x

[B56] Mäkinen H. Colin F. (1999). Predicting the number, death, and self-pruning of branches in Scots pine. Can. J. For. Res. 29, 1225–1236. doi: 10.1139/x99-065

[B57] Mäkinen H. Hein S. (2006). Effect of wide spacing on increment and branch properties of young Norway spruce. Eur. J. For. Res. 125, 239–248. doi: 10.1007/s10342-006-0115-9

[B58] Matsuo T. Bongers F. Martínez-Ramos M. van der Sande M. T. Poorter L. (2024). Height growth and biomass partitioning during secondary succession differ among forest light strata and successional guilds in a tropical rainforest. Oikos 2024, e10486. doi: 10.1111/oik.10486

[B59] Mérian P. Lebourgeois F. (2011). Size-mediated climate–growth relationships in temperate forests: A multi-species analysis. For. Ecol. Manage. 261, 1382–1391. doi: 10.1016/j.foreco.2011.01.019

[B60] Meteyard L. Davies R. A. I. (2020). Best practice guidance for linear mixed-effects models in psychological science. J. Memory Lang. 112, 104092. doi: 10.1016/j.jml.2020.104092

[B61] Nicolini E. Beauchêne J. de la Vallée B. L. Ruelle J. Mangenet T. Heuret P. (2012). Dating branch growth units in a tropical tree using morphological and anatomical markers: the case of Parkia velutina Benoist (Mimosoïdeae). Ann. For. Sci. 69, 543–555. doi: 10.1007/s13595-011-0172-1

[B62] Oboite F. O. Comeau P. G. (2020). The interactive effect of competition and climate on growth of boreal tree species in western Canada and Alaska. Can. J. For. Res. 50, 457–464. doi: 10.1139/cjfr-2019-0319

[B63] Osada N. (2006). Crown development in a pioneer tree, Rhus trichocarpa, in relation to the structure and growth of individual branches. New Phytol. 172, 667–678. doi: 10.1111/j.1469-8137.2006.01857.x 17096793

[B64] Osada N. (2011). Height-dependent changes in shoot structure and tree allometry in relation to maximum height in four deciduous tree species. Funct. Ecol. 25, 777–786. doi: 10.1111/j.1365-2435.2011.01833.x

[B65] Pach M. Soberka M. (2011). The application of a retrospective dynamic competition index to assess the impact of neighboring trees on silver fir (Abies alba Mill.) basal area increment. For. Res. Papers 72, 357–366. doi: 10.2478/v10111-011-0035-4

[B66] Pawson S. M. Brin A. Brockerhoff E. G. Lamb D. Payn T. W. Paquette A.. (2013). Plantation forests, climate change and biodiversity. Biodiversity Conserv. 22, 1203–1227. doi: 10.1007/s10531-013-0458-8

[B67] Piutti E. Cescatti A. (1997). A quantitative analysis of the interactions between climatic response and intraspecific competition in European beech. Can. J. For. Res. 27, 277–284. doi: 10.1139/x96-176

[B68] Prévosto B. Curt T. Gueugnot J. Coquillard P. (2000). Modeling mid-elevation Scots pine growth on a volcanic substrate. For. Ecol. Manage. 131, 223–237. doi: 10.1016/S0378-1127(99)00216-9

[B69] Primicia I. Imbert J. B. Traver M. C. Castillo F. J. (2014). Inter-specific competition and management modify the morphology, nutrient content and resorption in Scots pine needles. Eur. J. For. Res. 133, 141–151. doi: 10.1007/s10342-013-0753-7

[B70] Rahman L. Umeki K. Honjo T. (2014). Modeling qualitative and quantitative elements of branch growth in saplings of four evergreen broad-leaved tree species growing in a temperate Japanese forest. Trees 28, 1539–1552. doi: 10.1007/s00468-014-1064-4

[B71] Remphrey W. R. Powell G. R. (1984). Crown architecture of Larix laricina saplings: quantitative analysis and modelling of (nonsylleptic) order 1 branching in relation to development of the main stem. Can. J. Bot. 62, 1904–1915. doi: 10.1139/b84-260

[B72] Roy A. (2024). “Chapter 12 - Plantation forests, biodiversity, and economy,” in Biodiversity and Bioeconomy. Eds. Singh K. Ribeiro M. C. Calicioglu Ö. (Netherlands: Elsevier Besloten Vennootschap), 263–279.

[B73] Sánchez-Salguero R. Linares J. C. Camarero J. J. Madrigal-González J. Hevia A. Sánchez-Miranda Á.. (2015). Disentangling the effects of competition and climate on individual tree growth: A retrospective and dynamic approach in Scots pine. For. Ecol. Manage. 358, 12–25. doi: 10.1016/j.foreco.2015.08.034

[B74] Scott P. A. Hansell R. I. C. Erickson W. R. (1993). Influences of wind and snow on northern tree-line environments at Churchill, Manitoba, Canada. Arctic 46, 316–323. doi: 10.14430/ARCTIC1359

[B75] Shao Q. Huang L. Liu J. Kuang W. Li J. (2011). Analysis of forest damage caused by the snow and ice chaos along a transect across southern China in spring 2008. J. Geographical Sci. 21, 219–234. doi: 10.1007/s11442-011-0840-y

[B76] Shi J. Liu X. Xiang W. (2022). Does climate play a more important role than competition in modeling height to crown base of Larix principis-rupprechtii in northern China? For. Ecol. Manage. 526, 120564. doi: 10.1016/j.foreco.2022.120564

[B77] Smith S. H. Bell J. F. (1983). Using competitive stress index to estimate diameter growth for thinned douglas-fir stands. For. Sci. 29, 491–499. doi: 10.1093/forestscience/29.3.491

[B78] Sprugel D. G. (2002). When branch autonomy fails: Milton’s Law of resource availability and allocation. Tree Physiol. 22 15-16, 1119–1124. doi: 10.1093/treephys/22.15-16.1119 12414371

[B79] Stephenson N. L. Das A. J. Ampersee N. J. Bulaon B. M. Yee J. L. (2019). Which trees die during drought? The key role of insect host-tree selection. J. Ecol. 107, 2383–2401. doi: 10.1111/1365-2745.13176

[B80] Sun J. Wang M. Lyu M. Niklas K. J. Zhong Q. Li M.. (2019). Stem and leaf growth rates define the leaf size vs. number trade-off. AoB Plants 11, plz063. doi: 10.1093/aobpla/plz063 31777650 PMC6863467

[B81] Tang W. Liu S. Kang P. Peng X. Li Y. Guo R.. (2021). Quantifying the lagged effects of climate factors on vegetation growth in 32 major cities of China. Ecol. Indic. 132, 108290. doi: 10.1016/j.ecolind.2021.108290

[B82] Tezara W. Mitchell V. J. Driscoll S. D. Lawlor D. W. (1999). Water stress inhibits plant photosynthesis by decreasing coupling factor and ATP. Nature 401, 914–917. doi: 10.1038/44842

[B83] Tian Q. He Z. Xiao S. Peng X. Lin P. Zhu X.. (2024). Intra-annual stem radial growth of Qinghai spruce and its environmental drivers in the Qilian Mountains, northwestern China. Sci. Total Environ. 915, 170093. doi: 10.1016/j.scitotenv.2024.170093 38224885

[B84] Wang J. Jiang L. Yan Y. (2022a). The impacts of climate, competition, and their interactions on crown width for three major species in Chinese boreal forests. For. Ecol. Manage. 526, 120597. doi: 10.1016/j.foreco.2022.120597

[B85] Wang L. Li X. Wang H. (2019). Physicochemical properties, bioaccessibility and antioxidant activity of the polyphenols from pine cones of Pinus koraiensis. Int. J. Biol. Macromolecules 126, 385–391. doi: 10.1016/j.ijbiomac.2018.12.145 30576738

[B86] Wang Z. Zhang X. Zhang J. Chhin S. (2022b). Effects of stand factors on tree growth of Chinese fir in the subtropics of China depends on climate conditions from predictions of a deep learning algorithm: A long-term spacing trial. For. Ecol. Manage. 520, 120363. doi: 10.1016/j.foreco.2022.120363

[B87] Wang C. Zhao Z. Hein S. Zeng J. Schuler J. Guo J.. (2015). Effect of Planting Density on Knot Attributes and Branch Occlusion of Betula alnoides under Natural Pruning in Southern China. Forests 6, 1343–1361. doi: 10.3390/f6041343

[B88] Weber P. Bugmann H. Fonti P. Rigling A. (2008). Using a retrospective dynamic competition index to reconstruct forest succession. For. Ecol. Manage. 254, 96–106. doi: 10.1016/j.foreco.2007.07.031

[B89] Weiskittel A. R. Maguire D. A. Monserud R. A. (2007). Response of branch growth and mortality to silvicultural treatments in coastal Douglas-fir plantations: Implications for predicting tree growth. For. Ecol. Manage. 251, 182–194. doi: 10.1016/j.foreco.2007.06.007

[B90] Wilson B. F. (2000). Apical control of branch growth and angle in woody plants. Am. J. Bot. 87, 601–607. doi: 10.2307/2656846 10811784

[B91] Yan Y. Wang J. Mahardika S. B. Jiang L. (2023). Effects of climate and competition on crown width: a case of Korean pine plantations. Eur. J. For. Res. 142, 231–244. doi: 10.1007/s10342-022-01515-y

[B92] Yang J. Cooper D. J. Zhang X. Song W. Li Z. Zhang Y.. (2022). Climatic controls of Pinus pumila radial growth along an altitude gradient. New Forests 53, 319–335. doi: 10.1007/s11056-021-09858-x

[B93] Yang Y. Huang S. (2018). Effects of competition and climate variables on modelling height to live crown for three boreal tree species in Alberta, Canada. Eur. J. For. Res. 137, 153–167. doi: 10.1007/s10342-017-1095-7

[B94] Young D. J. N. Stevens J. T. Earles J. M. Moore J. Ellis A. Jirka A. L.. (2017). Long-term climate and competition explain forest mortality patterns under extreme drought. Ecol. Lett. 20, 78–86. doi: 10.1111/ele.12711 28000432

[B95] Yuan Y. Khourchi S. Li S. Du Y. Delaplace P. (2023). Unlocking the multifaceted mechanisms of bud outgrowth: advances in understanding shoot branching. Plants (Basel) 12, 3628. doi: 10.3390/plants12203628 37896091 PMC10610460

[B96] Zhang H. Zou P. Zhao H. Qiu J. Regenstein J. M. Yang X. (2021). Isolation, purification, structure and antioxidant activity of polysaccharide from pinecones of Pinus koraiensis. Carbohydr. Polymers 251, 117078. doi: 10.1016/j.carbpol.2020.117078 33142621

[B97] Zhao D. Bullock B. P. Montes C. R. Wang M. (2020). Rethinking maximum stand basal area and maximum SDI from the aspect of stand dynamics. For. Ecol. Manage. 475, 118462. doi: 10.1016/j.foreco.2020.118462

[B98] Zhou M. Lei X. Lu J. Gao W. Zhang H. (2022). Comparisons of competitor selection approaches for spatially explicit competition indices of natural spruce-fir-broadleaf mixed forests. Eur. J. For. Res. 141, 177–211. doi: 10.1007/s10342-021-01430-8

[B99] Zou X. Miao Z. Hao Y. Liu X. Dong L. Li F. (2024). Effects of tree vigor, competition and stand conditions on branch diameter for mixed plantations of Fraxinus mandshurica Rupr. and Larix olgensis Henry in Northeast China. Eur. J. For. Res. 143, 1165–1180. doi: 10.1007/s10342-024-01681-1

